# Optimizing energy-efficient grid performance: integrating electric vehicles, DSTATCOM, and renewable sources using the Hippopotamus Optimization Algorithm

**DOI:** 10.1038/s41598-024-79381-4

**Published:** 2024-11-22

**Authors:** M. A. Abdelaziz, A. A. Ali, R. A. Swief, Rasha Elazab

**Affiliations:** 1 Electrical Power and Machines Department, Helwan University, Cairo, Egypt; 2 Renewable Electrical Energy Department, The British University in Egypt, El-Sherouk, Egypt; 3 Electrical Power and Machine Department, Ain Shams University, Cairo, Egypt

**Keywords:** Electric vehicles charging stations, Photovoltaic integration, DSTATCOM, Voltage stability, Power losses, Economic analysis, Energy grids and networks, Power distribution

## Abstract

The rapid increase in renewable energy integration and electric vehicle (EV) adoption creates significant challenges for the stability and efficiency of power distribution networks. This study addresses the need for optimized placement and sizing of Electric Vehicle Charging Stations (EVCSs), photovoltaic (PV) systems, and Distribution Static Compensators (DSTATCOMs) to enhance grid performance. The motivation for this work arises from the fluctuating nature of renewable energy generation and the unpredictable demands of EV charging, which strain existing infrastructure. To address these challenges, we propose a novel optimization framework that introduces the Renewable Distributed Generation Hosting Factor (RDG-HF) and Electric Vehicle Hosting Factor (EV-HF) as key metrics. These metrics, combined with the Hippopotamus Optimization Algorithm (HO), enable strategic planning within the IEEE 69-bus system. Simulation results demonstrate that the integrated placement of EVCSs, PVs, and DSTATCOMs reduces power losses by up to 31.5% and reactive power losses by up to 29.2%. An economic analysis further reveals payback periods ranging from 2.7 to 10.4 years and potential profits of up to $1,052,365 over 25 years. These findings highlight the importance of optimized integration in improving both technical performance and long-term economic benefits for distribution networks.

## Introduction

The increasing adoption of electric vehicles (EVs) necessitates the integration of electric vehicle charging stations (EVCSs) into distribution networks, which poses significant challenges. Extensive research has focused on optimizing EVCS placement from an operational perspective, considering factors such as waiting time, driving range, user satisfaction, and its relationship with market size^1,2^. However, a critical gap remains in understanding the impact of the EV demand load on distribution networks. Efforts have aimed at minimizing power losses and voltage deviations while considering hosting factors (HFs). Notably, current research primarily focuses on EV hosting factors (EV-HF) and renewable distributed generation hosting factors (RDG-HF). This introduction highlights recent research efforts to optimize EVCSs within distribution networks and underscores the need for further exploration of the relationship between EV demand load and network performance.

Considerable research has been devoted to optimizing EVCS placement, emphasizing factors such as waiting time, driving range, and user satisfaction^3–8^. Despite this, there is a pressing need to examine the impact of the EV demand load on distribution networks, considering both EV-HF and RDG-HF.

Previous studies have primarily concentrated on reducing active and reactive power losses for various EV-HF values. For instance, the Quantum-Behaved Gaussian Mutational Dragonfly Algorithm (QGDA) has been employed for optimization^9^. However, these studies often overlook the influence of RDG-HF. Algorithms such as Harmony Particle Swarm Optimization (PSO) have demonstrated improvements in voltage quality without accounting for RDG-HF^10,11^.

Efforts to enhance the integration of EVCSs with other network components, such as Distributed Generators (DGs) and DSTATCOMs, aim to improve network efficiency and reliability^12–18^. Nonetheless, the limited consideration of EV-HF and RDG-HF complicates the evaluation of these efforts. For example, in^19^, an RDG-HF exceeding 60% potentially surpassed the limits set by specific countries for Radial Distribution Networks (RDNs)^20,21^.

A distinct research direction involves optimizing the power loss within distribution networks that incorporate both EVCSs and RDGs^12–18,22–24^. Techniques such as an AI-based approach using the hybrid of grey wolf optimization and particle swarm optimization, Modified Teaching-Learning-Based Optimization (TLBO), and Multi-Objective Particle Swarm Optimization (MOPSO) have been utilized. However, these studies lack a comprehensive investigation into the interaction and influence of hosting factors on network performance, highlighting a significant research gap.

Several studies have explored minimizing energy loss and voltage deviation using various optimization techniques, including Differential Evolution (DE), Grey Wolf Optimizer (GWO), adaptive particle swarm optimization, and Fuzzy Analytic Hierarchy Process (AHP)^25–33^. Regrettably, these studies often neglect the proper use of RDG-HF, thereby limiting the scope of their findings. In^28^, the EV-HF is assumed to be equal to the total demand load. A comprehensive review of optimal electric vehicle charging station placement, analyzing various objective functions, solution techniques, and geographic conditions to improve the planning of charging infrastructure in smart grids^34^.

In contrast, numerous studies addressing uncertainties in EV-HF through probabilistic modeling tend to overlook the role of RDG-HF^35–41^. Additionally, integrating renewable sources at charging stations, which reduces grid demand, frequently ignores RDG-HF^42–46^. This raises questions about its applicability in scenarios with high renewable energy penetration. Most studies focus on minimizing active power loss and voltage deviation using optimization algorithms such as GWO-PSO^38,43^, Chicken Swarm Optimization (CSO), and TLBO^44^, a general algebraic modeling system^34^, and a mixed optimization of Lazy Greedy with Direct Gain and Lazy Greedy with Effective Gain (LGEG)^46^. This emphasis might lead to the neglect of other critical network performance attributes under high RDG-HF conditions.

Various optimization techniques have been employed to determine the optimal placement of EVCSs, aiming to minimize energy loss and enhance reliability. Methods include Mixed Integer Nonlinear Programming (MINLP)^47^, Differential Evolution combined with Harris Hawks Optimization (DE-HHO)^48^, the Binary Atom Search algorithm^49^, and the BAT algorithm^50^. However, these approaches often disregard RDG-HF, limiting the optimization scope. Notably, EV-HF is typically presented with discrete values, such as 5%, 10%, 15%, and 20%^50^.

Alternatively, few studies have investigated the problem from the standpoint of communication efficiency in charging stations and economic dispatch^51–53^.

While energy storage systems are widely recognized for their ability to balance the variability of renewable energy production and mitigate the mismatch between supply and demand^54^, this study focuses on a robust optimization approach that addresses grid stability without the inclusion of storage. The proposed framework demonstrates that through the optimized integration of EVCSs, PV, and DSTATCOMs, it is possible to significantly improve grid performance under stochastic conditions. The robustness of the model ensures effective system operation, even without storage, offering a novel perspective on planning for future grid integration challenges. Future work may extend this framework to incorporate storage systems for further optimization and flexibility.

**Fig. 1:**
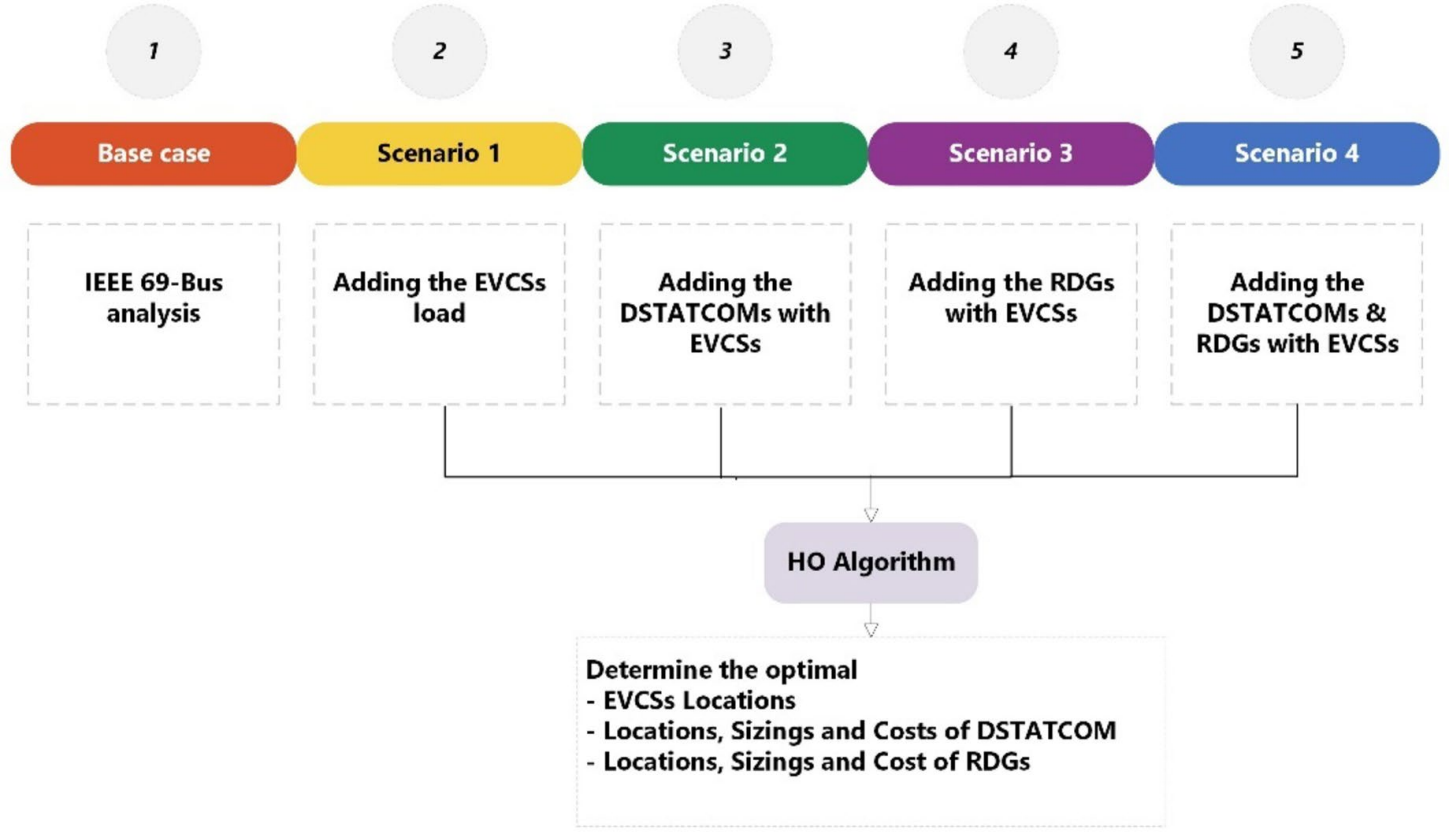
The study graphical framework.

While many studies acknowledge variations in EV-HF distribution^55–58^, they rarely specify the RDG-HF percentage, except in^59^, where RDG-HF exceeded 40%. This highlights the need for more explicit consideration of RDG-HF in future research. In this study, the impact of EV-HF and Renewable Distributed Generation Hosting Factor RDG-HF is examined across five different scenarios involving the integration of EVCSs and DSTATCOMs. The summarization and all the scenarios of the study are shown in Fig. 1.

The limitations of previous studies can be summarized as follows: Many previous studies have focused solely on the impact of DSTATCOM or RDGs, without accounting for the influence of EV loads. While a few studies have examined the effect of EV loads, they often overlook the hosting factor, instead representing EV loads as a simple count of vehicles within the distribution network. Moreover, most research incorporating RDGs fails to consider the maximum allowable hosting factor of RDGs in relation to the total load capacity of the distribution network.

The contributions of the study can be summarized as follows:

- Investigates the impact of EVCSs on the performance of a RDN under four distinct scenarios, each considering different EV-HFs and RDG-HFs values.
- Integrates the constraints and hosting capacities of RDG to ensure the network operates within safe and efficient limits, optimizing the placement of both renewable energy sources and compensators.
- Considers both technical and economic aspects by evaluating the investment cost and payback period for DSTATCOMs and RDGs. The payback period is critical for determining long-term feasibility, with values ranging from 2.7 to 10.4 years based on the scenario.
- Utilizes a multi-objective optimization function to achieve a comprehensive techno-economic solution. The function minimizes power losses, voltage deviations, and installation costs while maximizing voltage stability and annual savings. This optimization ensures the optimal placement of EVCSs and the sizing and location of DSTATCOMs and RDGs within the IEEE 69-bus system.
- Introduces a novel approach leveraging the Hippopotamus Optimization Algorithm to balance network efficiency, stability, and economic benefits, demonstrating up to a 31.5% reduction in power losses and highlighting a potential profit of over $1 million over 25 years.

This contribution effectively addresses the critical challenges posed by increasing electric vehicle adoption and renewable energy integration, offering practical insights into optimal infrastructure planning for future smart grids.

## Problem formulation

The surge in EV consumption presents a critical challenge for both EV customers and Distribution Network Operators (DNOs). EV customers are concerned with the distance to charging stations (CSs), while DNOs face the impact of EV charging, which can lead to increased real and reactive power losses and voltage drops on the buses of radial distribution systems (RDSs). Proper positioning of EVCSs is vital for minimizing power losses and enhancing voltage stability by incorporating Renewable Distributed Generators (RDGs).

This study proposes a novel approach for hosting renewable energy, such as solar energy, in the RDS with various hosting percentages. The RDG systems are paired with compensators (DSTATCOMs) to mitigate the effect of the EV hosting factor (HF-EV). The HF-RDG is a percentage of the maximum demand load of the RDS^20^. This study aims to evaluate the performance of the RDS under different HF-EV values, focusing on minimizing active and reactive power losses and boosting the voltage stability index.

## Methodology

### PV/DSTATCOM technical model

RDG and DSTATCOMs are modeled by their respective contributions of injected active and reactive power to the electrical grid. The active power injected by RDGs, primarily photovoltaic PV systems, and DSTATCOMs are determined based on their respective capacities, which are established through a detailed sizing process, as discussed in^60–62^.

### RDG and DSTATCOM sizing

Through the utilization of RDGs and DSTATCOMs, grid stability is provided. The maximum capacity of RDGs must be within the given limit of the DNO^20^, despite their high potential. The maximum capacity of the RDG in each zone ($\:{P}_{\text{m}\text{a}\text{x}\_zone}^{RDG}$) can be defined by multiplying the hosting factor ($\:HF$) by the cumulative power of the zone ($\:{P}_{Demand}^{Zone}$), as shown in Eq. (1)^20^.

The Hosting Factor (HF) represents the capacity of a radial distribution network to integrate additional load or generation without negatively impacting its stability, reliability, or requiring substantial upgrades to the system. Specifically, HF is the ratio, typically expressed as a percentage, of the maximum permissible load or generation that can be connected to the network under current infrastructure constraints, relative to the total load demand. This factor reflects the network’s ability to manage the integration of new loads or distributed energy resources, while maintaining voltage levels, thermal limits, and ensuring system reliability. The calculation of HF typically considers a variety of technical parameters that differ according to the studied grid.

1 $$\:{P}_{\text{m}\text{a}\text{x}\_zone}^{RDG}=HF\times\:{P}_{Demand}^{Zone}$$

Likewise, the varying effective ratings of RDGs ($\:{P}_{zone}^{RDG}$) should fall between the minimum ($\:{P}_{\text{m}\text{i}\text{n}\_zone}^{RDG}$) and maximum ($\:{P}_{\text{m}\text{a}\text{x}\_zone}^{RDG}$) ratings. Additionally, the reactive power ($\:{Q}^{DSTATCOM}$) should be kept within the working range of DSTATCOMs, where ($\:{Q}_{min}^{DSTATCOM}$) and ($\:{Q}_{max}^{DSTATCOM}$) are the upper and lower working ranges of DSTACOMs, respectively. Equations (2) and (3) illustrate the working ranges of RDGs and DSTATCOMs^60–62^.

2 $$\:{P}_{\text{m}\text{i}\text{n}\_zone}^{RDG}\le\:{P}_{zone}^{RDG}\le\:\:{P}_{\text{m}\text{a}\text{x}\_zone}^{RDG}$$

3 $$\:{Q}_{min}^{DSTATCOM}\le\:{Q}^{DSTATCOM}\le\:\:{Q}_{max}^{DSTATCOM}$$

### Cost analysis

#### DSTATCOM cost

The annual investment cost ($$\:{AC}_{DSTATCOM}$$) of the DSTATCOM can be calculated using the formula provided in Eq. (4), where $$\:{C}_{DSTATCOM}$$​ represents the cost of the DSTATCOM, $$\:{B}_{D}$$ is the rate of return, and $$\:{n}_{D}$$ is the operational lifetime of the DSTATCOM in years. For this analysis, we assume $$\:{C}_{DSTATCOM}$$​=50 $/kVAr, $$\:{n}_{D}$$=1 year, and $$\:{B}_{D}$$=0.1^63^.

4 $$\:{AC}_{DSTATCOM}={C}_{DSTATCOM}\frac{{\left(1+{B}_{D}\right)}^{{n}_{D}}\:\times\:\:{B}_{D}}{{\left(1+{B}_{D}\right)}^{{n}_{D}}-1}$$

In this context, the total annual cost savings ($$\:TACS$$) are determined by considering the overall energy loss costs before and after the installation of the DSTATCOM. This can be calculated using Eq. (5), where $$\:{K}_{{P}_{loss}}$$ is the energy cost of losses (given as 0.06 $/kWh), $$\:T$$ represents the total annual hours (8760 h), and $$\:{P}_{loss}^{Before}$$​ and $$\:{P}_{loss}^{After}$$​ are the total active power losses before and after the installation of the DSTATCOM, respectively^63^.

5 $$\:TACS={K}_{{P}_{loss}}\left(T\times\:{P}_{loss}^{Before}\right)-{K}_{{P}_{loss}}\left(T\times\:{P}_{loss}^{After}\right)-{AC}_{DSTATCOM}$$

The total annual cost savings in the per-unit system can then be expressed through Eq. (6), incorporating the specifics of the power losses and energy costs into a comprehensive financial analysis^63^.

6 $$\:{TACS}_{p.u}=\frac{TACS}{{K}_{{P}_{loss}}\left(T\times\:{P}_{loss}^{Before}\right)}$$

These equations provide a structured method for evaluating the financial benefits of DSTATCOM implementation, allowing for an accurate assessment of both the initial investment and the potential cost savings over time. This methodology is crucial for ensuring the economic feasibility and justification of deploying DSTATCOMs in power systems.

#### PV cost

The total cost of PV ($\:{PV}_{Total\_Cost}$) can be broken down into many costs, such as solar modules, solar inverters, structural balance of system (BOS), electrical balance of system (BOS), installation of labor and equipment, contractor overhead, sales tax, permitting, inspection and interconnection (PII), transmission line costs, developer overhead, contingency budget, and contractor and developer profit^64^. Therefore, the total cost of the PV distributed generator is calculated via Eq. (7).

7 $$\:{PV}_{Total\_Cost}={C}_{PV/W}\times\:{PV}_{size}$$

where$\:{C}_{PV/W}$is the total cost of PV per watt and$\:{PV}_{size}$is the size of the PV.

### Technical modeling of the system

The technical modeling sector can be divided into power balance, voltage limits, reactive power limitations of DSTATCOM, and real power limitations of RDG. The locations of the RDG and DSTATCOM are essential parameters in the power equations of the RDS.

#### Power balance

The power balance constraints are expressed as follows:

8 $$\:{P\:}_{Total\_Loss}+\:\sum\:{P}_{m}^{Demand}\:+\:\sum\:{P}_{m}^{EVCS}\:=\:\sum\:(\:{P}_{m}^{DSTATCOM}\:+\:{P}_{m}^{RDGs})$$

The variables $\:{P\:}_{Total\_Loss},\:{P}_{m}^{Demand},\:{{P}_{m}^{EVCS},\:P}_{m}^{DSTATCOM}$ and $\:{P}_{m}^{RDGs}$ indicate the total power loss in the RDS, the total demand of the RDS, the total demand of the EVCSs, the power of the DSTATCOM, and the power injected by the RDGs, respectively.

#### Voltage limit

The voltage limits at the m-th bus in the RDS are given by:

9 $$V_{m}^{{\hbox{min} }} \leq {V_m} \leq V_{m}^{{\hbox{max} }}$$

where $\:{V}_{m}^{min}$ and $\:\:{V}_{m}^{max}$ are the lower and upper limits of the bus voltage, respectively.$\:{V}_{m}.$

#### Reactive power compensation

The limit of reactive power compensation DSTATCOM is denoted in Eq. (10), where $\:{Q}_{DSTATCOM\left(m\right)}^{min}$ and $\:{Q}_{DSTATCOM\left(m\right)}^{MAX}$ are the lower and upper limits of the DSTATCOM reactive power, respectively.

10 $$\:{Q}_{DSTATCOM\left(m\right)}^{min}\le\:{Q}_{DSTATCOM\left(m\right)}\le\:\:{Q}_{DSTATCOM\left(m\right)}^{MAX}$$

#### Real power compensation by RDG

RDGs exhibit limits dictated by the RDS characteristics and the geographic region within each country. HF-RDGs vary across nations. For instance, in Egypt, the HF-RDG is established at 1.5% of the maximum demand load of the RDS. Conversely, in Portugal, the upper threshold for HF-RDG stands at 25%, while in South Africa, it is specified not to surpass 15%^20^.

The RDG must ensure that the power injected at each optimized bus falls within the specified minimum and maximum limitations. The maximum RDG power of each zone is indicated in Eq. (1). Equation (2) specifies the specific quantity of real power adjustment that RDG $\:{P}_{RDG\left(zone\right)}$ provides for the system in each zone.

#### Voltage deviation index (VDI)

One of the objectives is to minimize the voltage deviation index. Voltage deviation refers to the difference between the nominal voltage and the measured value. The closer the bus voltage is to the nominal voltage, the better the voltage condition of the system. The calculation of the VDI is displayed in Eq. (11)^65^, where $\:{V}_{i}$and $\:{V}_{Ni}$are the voltage and nominal voltage at the $\:{i}^{th}$ node, respectively.

11 $$\:VDI=\sum\:_{i}^{Ni}\left|{V}_{n}-{V}_{i}\right|$$

#### Voltage Stability Index (VSI)

Various criteria are applied to evaluate the safety level of power systems. This research offers a Voltage Stability Index (VSI) designed for steady-state settings aimed at detecting nodes with heightened sensitivity to voltage collapse. Derived from power flow analysis, the index, abbreviated as VSI and expressed by Eq. (12)^66^, serves to determine the stability of the voltage at each node. For stable operation of an RDS, the VSI should be equal to or greater than zero (m ≥ 0). Nodes with lower VSI values imply a greater need for compensators to provide voltage stability.

12 $$\:VSI\left(m+1\right)={\left|{V}_{m+1}\right|}^{4}-4\:{\left[{P}_{m,m+1}\:{X}_{m,m+1}-{Q}_{m,m+1}\:{R}_{m,m+1}\right]}^{2}-4\left[{P}_{m,m+1}\:{R}_{m,m+1}+{Q}_{m,m+1}\:{X}_{m,m+1}\right]\:{\left|{V}_{m,m+1}\right|}^{2}$$

$\:{V}_{m+1}\:$ denotes the voltage magnitude at the $\:{(m+1)}^{th}$ bus, while $\:{X}_{m,m+1},\:\:{R}_{m,m+1},{Q}_{m,m+1}$ and $\:\:{P}_{m,m+1}$ refer to the resistance, reactance, reactive power flow, and real power flow, respectively, of the line connecting the $\:{m}^{th}$ and $\:{(m+1)}^{th}$ buses.

#### The objective function

The aim of this study is to determine the optimal locations of EVCSs, DSTATCOMs, and RDGs. The optimal locations are the locations that achieve the minimum active power loss (P_loss_), reactive power loss (Q_loss_), voltage deviation index (VDI), installation cost of PVs or DSTATCOMs (C_instal_), and total annual savings (TAS) while improving the VSI. The following equation presents the weighted fitness function to determine the optimal solution. where $\:{w}_{1}$, $\:{w}_{2}$, $\:{w}_{3}$,$\:\:{w}_{4}$, $\:{w}_{5}$, and $\:{w}_{6}$ are the weight factors used to equilibrate the fitness function.

13 $$\:Fit\:fun={w}_{1}\:{P}_{loss}+{w}_{2}\:{Q}_{loss}+{w}_{3}\:\text{V}\text{D}+{w}_{4}\:VSI+{w}_{5}\:{C}_{install}+{w}_{6}\:TAS$$

## Hippopotamus optimization algorithm

The Hippopotamus Optimization (HO) algorithm is a bioinspired metaheuristic optimization technique modeled after the social behavior and defense mechanisms of hippo herds. It has been compared with 12 other popular metaheuristic algorithms by the benchmark functions, including the Whale Optimization Algorithm (WOA), Gray Wolf Optimization (GWO), and Particle Swarm Optimization (PSO), consistently outperforming them in finding global optima and avoiding local traps. Its superior performance across several benchmark functions and real-world engineering problems further validates its effectiveness^67^. The HO algorithm consists of three phases:

### Phase 1: Hippopotamuses position update in the river or pond (exploration)

This phase aims at exploring the search space similarly to how hippos go about their environment, the water. Therefore, the movement and location of individual males ($\:{{x}_{ij}}^{M\:hippo}$), females, and the dominant hippo ($\:{D}^{hippo}$) control the exploration process. From Eq. (14), the dominant hippo (representing the current best solution) moves other individuals in relation to its distance from it. This distance is, in turn, a function of not only the dominance of the hippo but also of a random vector ($\:{x}_{ij}$). ($\:{\chi\:}_{i}$), and ($\:{y}_{1}$) and an integer ($\:{I}_{1}$) representing inherent variability observed in exploration^67^.

14 $$\:{{\upchi\:}}_{i}^{M\:hippo}:{{x}_{ij}}^{M\:hippo}={x}_{ij}+{y}_{1}\cdot\:\left({D}^{hippo}-I1{x}_{ij}\right)$$

If a male or female hippopotamus’s position results in a superior objective function value compared to the current dominant hippopotamus, the dominant’s position is then replaced with that individual’s position. This mechanism ensures that the exploration process continuously seeks better solutions.

### Phase 2: the defense action of Hippopotamus against predators (exploration)

The defense mode comes into play when something to defend against (e.g., crocodiles of the Nile) is detected by the herd. Immediate defense from the predator is undertaken, accompanied by loud vocalization. Equation (15) can be used to model the rapid turn toward the threat. where the random movement of predators ($\:{\mathcal{P}\text{\:redator}}_{j}$) is denoted by a random vector ($\:{\overrightarrow{r}}_{8}$) ranging from zero to one within the upper ($\:{ub}_{j}$) and lower ($\:{ll}_{j}$) limits of the decision variables at ($\:{j}^{th}$). Additionally, Eq. (16) models the change in distance between predator and hippo after the defense response^67^.

15 $$\:\text{Predator\::\:}{\mathcal{P}\text{\:redator}}_{j}\text{\:}={ll}_{j}+{\overrightarrow{r}}_{8}\cdot\:\left({ub}_{j}-{ll}_{j}\right),\:j=\text{1,2},\dots\:,m$$

16 $$\:\overrightarrow{\mathcal{D}}=\left|{\mathcal{P}\text{\:redator}}_{j}\text{\:}-{x}_{i\varvec{j}}\right|$$

### Phase 3: Hippopotamus escaping from the predator (exploitation)

The hippos may escape overwhelming predator attacks or situations in which they cannot mount a sufficient defense to more protected areas. Mathematically, these stochastic refugia are modeled to simulate the unpredictability of escape routes. Since a new location provides a better objective function value (indicating a better solution), this would characterize how a hippo escapes, symbolizing a successful escape.

**Fig. 2:**
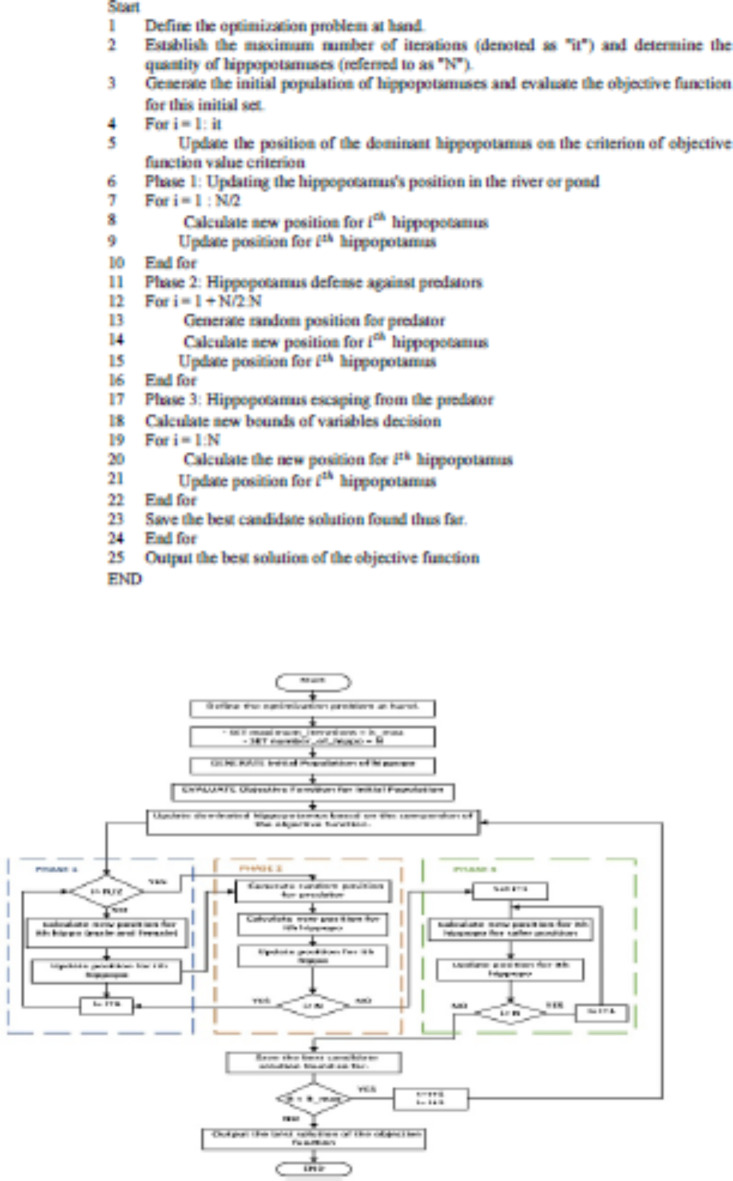
The Flowchart of HO algorithm.

The HO algorithm is a sophisticated method that considers the complexities of optimization problems and devises an efficient search strategy by mimicking the behavior of hippos. Throughout successive iterative phases simulating herd dynamics and defensive mechanisms, the algorithm proves to be robust and efficient for most optimization problems. The following is the pseudocode and flowchart describing the HO algorithm in Fig. 2.

## Results and discussion

This study investigates the impact of increasing electric vehicle (EV) adoption and renewable energy sources on the performance of a local power grid, specifically a Radial Distribution Network (RDN). The Renewable Distributed Generation Hosting Factor (RDG-HF) and the EV Hosting Factor (EV-HF) are used to represent these variables.

The analysis is conducted using the IEEE 69-bus system, which represents a scenario with 3.80 MW of active power and 2.69 MVAR of reactive power, as shown in Fig. 1a. Five distinct scenarios are explored to understand the effects of varying EV-HF and RDG-HF values, simulating the integration of the latest EV and renewable energy technologies into the RDN.

**Fig. 3:**
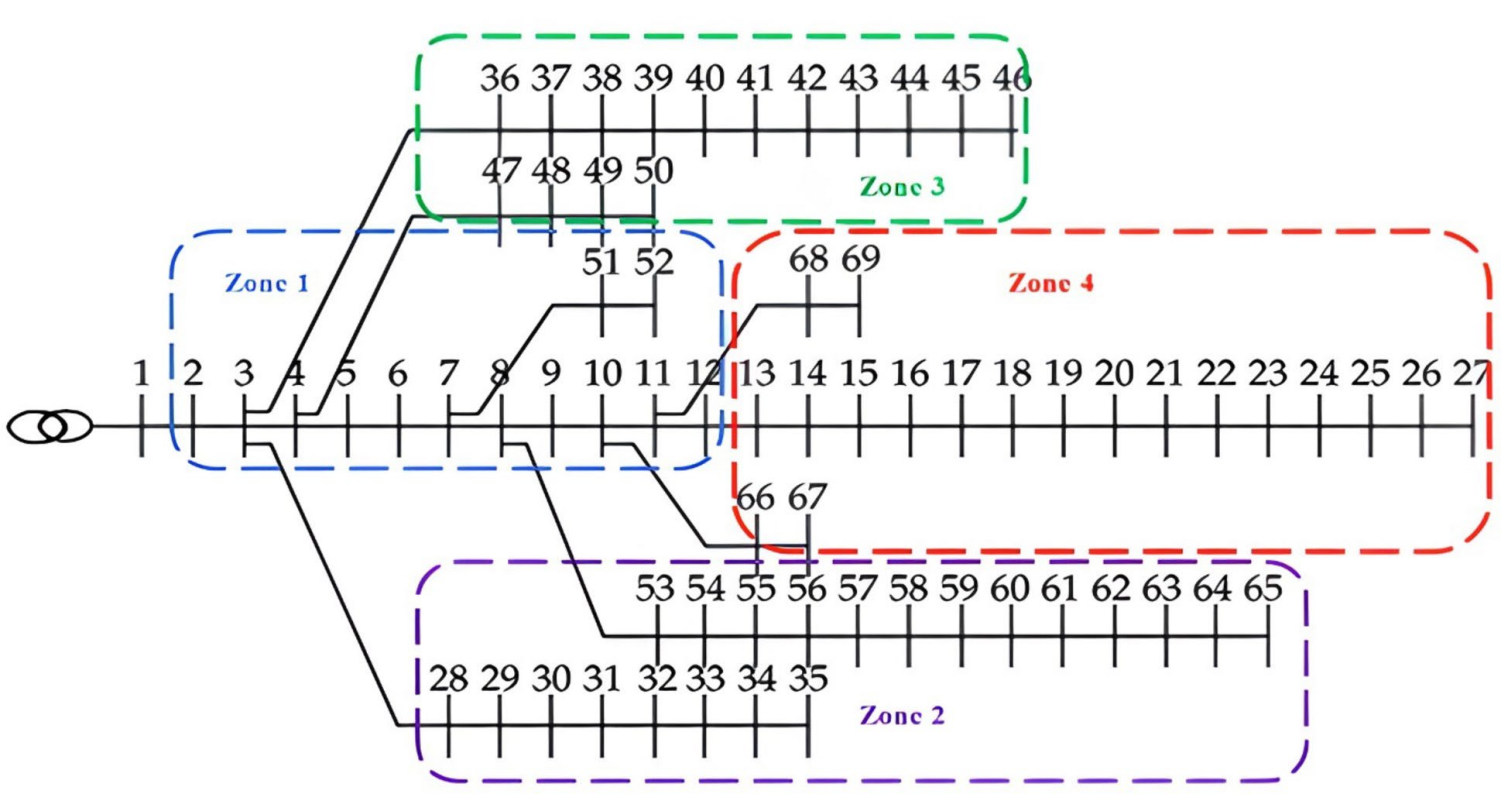
The clustering of the RDN.

The RDN is divided into four zones, each with a specific number of buses. The EV-HF in each zone is a ratio of the total demand within that zone, as detailed in Table 1; Fig. 3.

**Table 1:** The number of buses included in each zone and the sizes of EVCSs in each zone at different EV-HFs.

	Buses at each zone	Total demand load (kW)	EVCS demand load due to EV-HF (kW)			
			30%	40%	50%	60%
Zone 1	2-3-4-5-6-7-8-9-10-11-12-51-52	510.1	153.03	204.04	255.05	306.06
Zone 2	28-29-30-31-32-33-34-35-53-54-55-56-57-58-59-60-61-62-63-64-65	1808.2	542.48	723.30	904.13	1084.95
Zone 3	36-37-38-39-40-41-42-43-44-45-46-47-48-49-50	1034	310.21	413.62	517.02	620.42
Zone 4	13-14-15-16-17-18-19-20-21-23-24-25-26-27-66-67-68-69	393.8	118.14	157.52	196.90	236.28

### Scenario 1: integrating EVCSs with the RDN

**Fig. 4:**
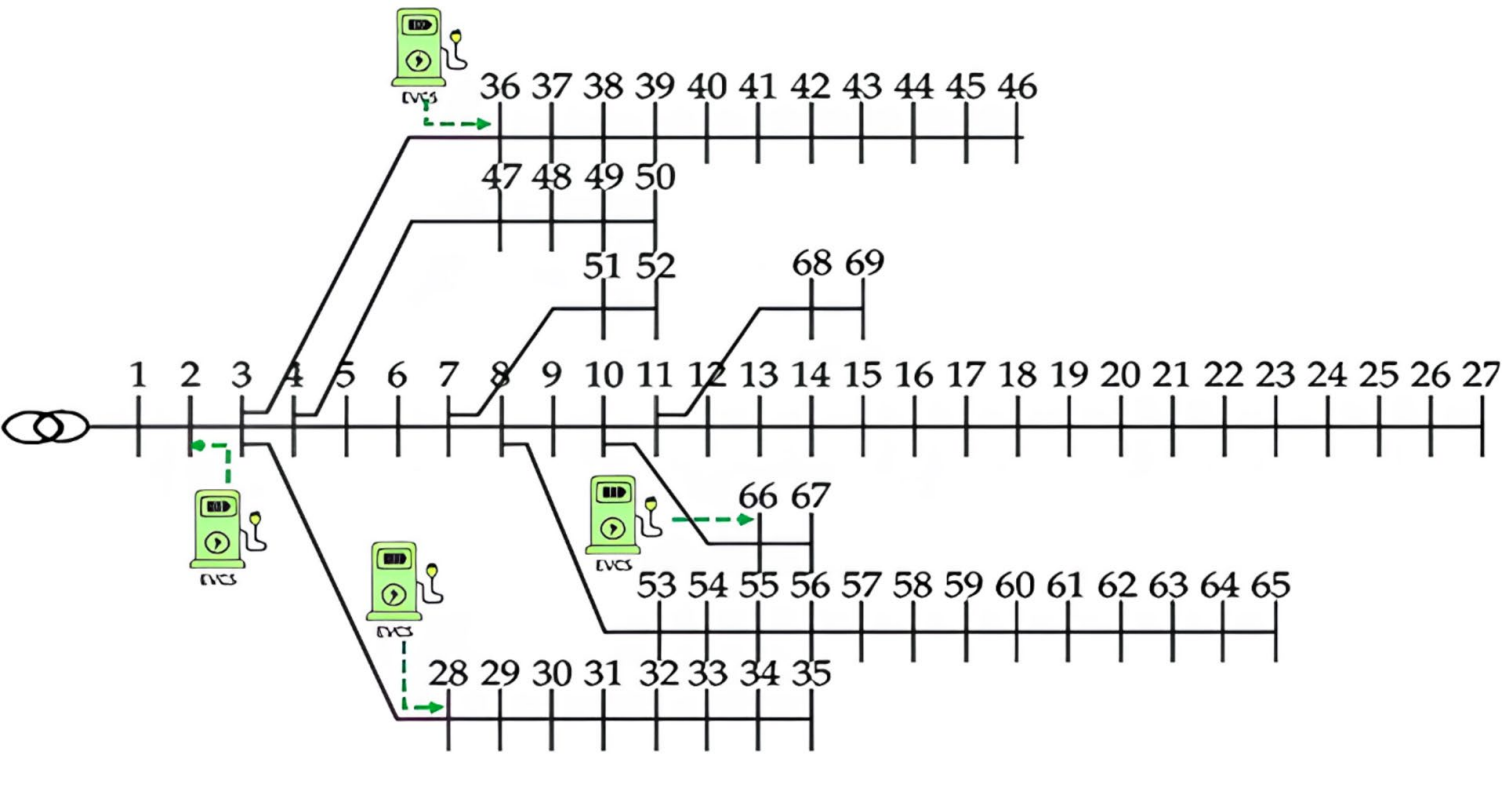
The optimal locations of EVCSs.

In this scenario, the optimal placement of Electric Vehicle Charging Stations (EVCSs) in each zone is determined using the HO algorithm. The EV-HF values considered are 0.3, 0.4, 0.5, and 0.6. The size of each EVCS is calculated based on the EV-HF and the total demand load of each zone, as shown in Table 1. The optimal locations for the EVCSs are at buses 2, 28, 36, and 66, as illustrated in Fig. 4.

As EV-HF increases, both the total active and reactive power increase, while the VSI, voltage deviation, and minimum voltage decrease compared to those in the base case, as shown in Table 4. The voltage profile and power losses across buses for different EV-HF values are illustrated in Figs. 6 and 7.

### Scenario 2: integration of EVCS and DSTATCOM

The HO algorithm is utilized to ascertain the optimal sizing and placement of DSTATCOMs in various zones, as well as the ideal locations for EVCSs. The integration of DSTATCOMs is intended to counteract the negative impacts of elevated EV-HF on power loss and voltage stability. The findings reveal significant reductions in active power losses (between 27% and 30%) and reactive power losses of 28% in comparison to the baseline scenario. Relative to the initial scenario, the reductions are approximately 31% for both active and reactive power losses. Additionally, there is a decrease in the voltage deviation index, while improvements are observed in the minimum voltage levels and the Voltage Stability Index (VSI) at all EV-HF levels. These effects are detailed in Table 2, which highlights the impact of DSTATCOMs on these metrics. Figures 6 and 7 illustrate the voltage and power loss profiles of scenario 2, providing a comparison with the baseline case.

**Table 2:** The impact of EV-HF on the objectives in scenario 2.

	Base	Scenario 2 EVCSs & DSTATCOMs			
EV-HF	–	30%	40%	50%	60%
$\:{P}_{loss}$ (kW)	225	155.44	156.54	158.46	162.97
$\:{Q}_{loss}$ (kVAR)	102.2	72.17	72.83	73.65	75.63
$\:VSI$	0.6868	0.7449	0.7451	0.7443	0.7389
VDI	0.0993	0.0594	0.0593	0.0609	0.0652
$\:{V}_{min}$ (pu)	0.9092	0.9279	0.9280	0.9277	0.9260

Table 3 outlines the results obtained through the HO algorithm, which identifies the optimal sizes and placements of DSTATCOMs across different zones. The sizes and locations of the DSTATCOMs vary due to the differing HFs in each zone. The average installation cost of DSTATCOMs across various EV-HF levels is estimated at $94,000, with annual savings fluctuating based on the EV-HF, as shown in Table 3.

**Table 3:** DSTATCOMs’ specifications in scenario 2.

	Zone	Scenario 2 (EVCSs & DSTATCOM)			
		EV-HF			
		0.3	0.4	0.5	0.6
DSTATCOM size (KVAR)	Zone 1	303	549	433	0
	Zone 2	1000	1000	1000	1000
	Zone 3	0	0	0	0
	Zone 4	597	428	541	550
DSTATCOM location (bus)	Zone 1	8	11	9	-
	Zone 2	61	61	61	61
	Zone 3	–	–	–	–
	Zone 4	16	20	17	21
Total DSTATCOMs cost ($)		95,050	99,200	100,450	80,750
Annual savings costs ($)		36560.6121	35983.66	34973.64	32600.68

### Scenario 3: the integration of EVCSs and RDGs

This scenario maintains the same EVCS size and location as in Scenario 1. The HO algorithm is used to determine the optimal locations and sizes of Renewable Distributed Generators (RDGs) in the IEEE 69-bus RDN, considering the RDG-HF restricted limitations (not exceeding 25%)^20^.

**Fig. 5:**
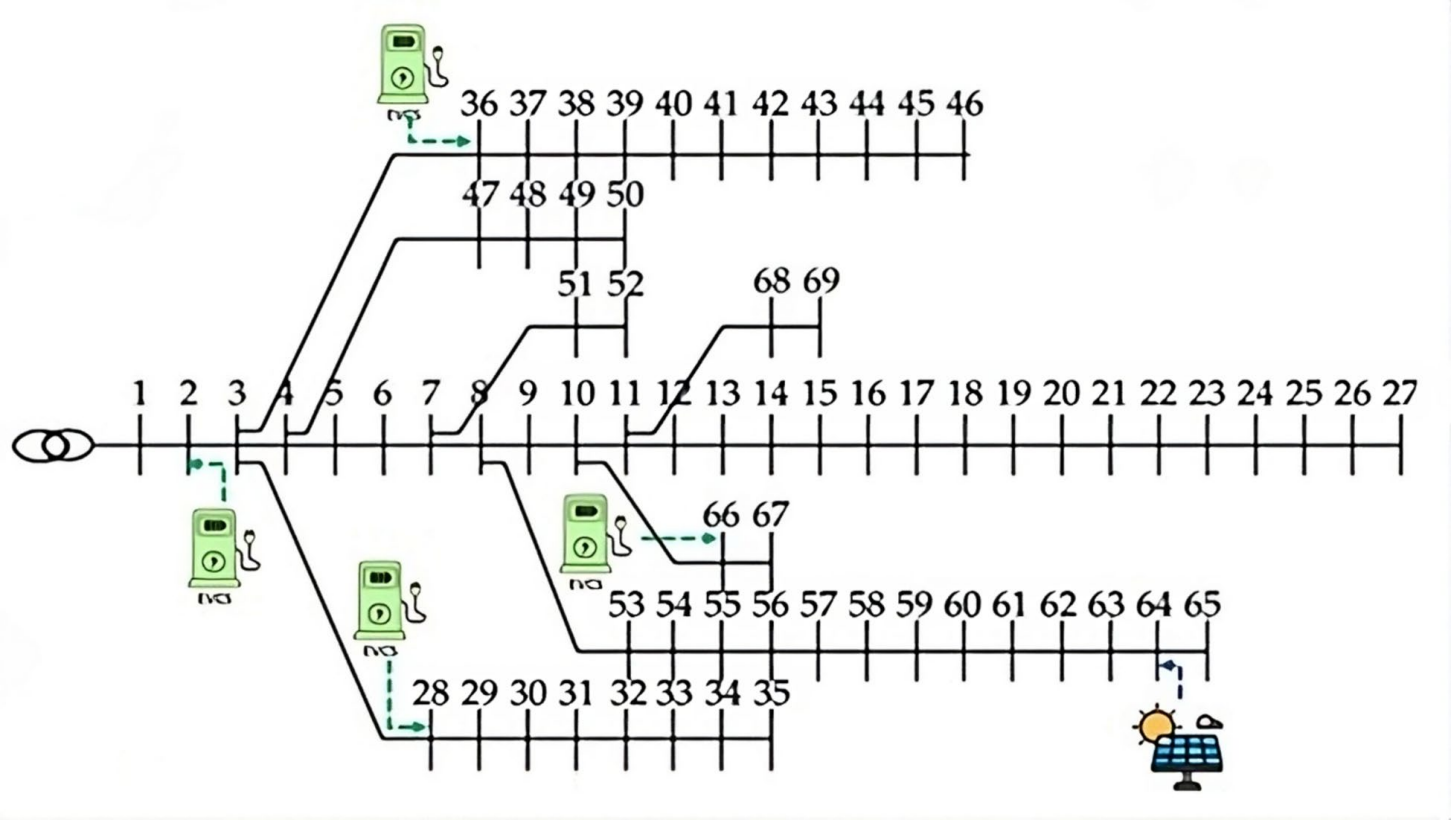
The proposed IEEE 69-bus as RDN in various conditions in scenario 3.

**Fig. 6:**
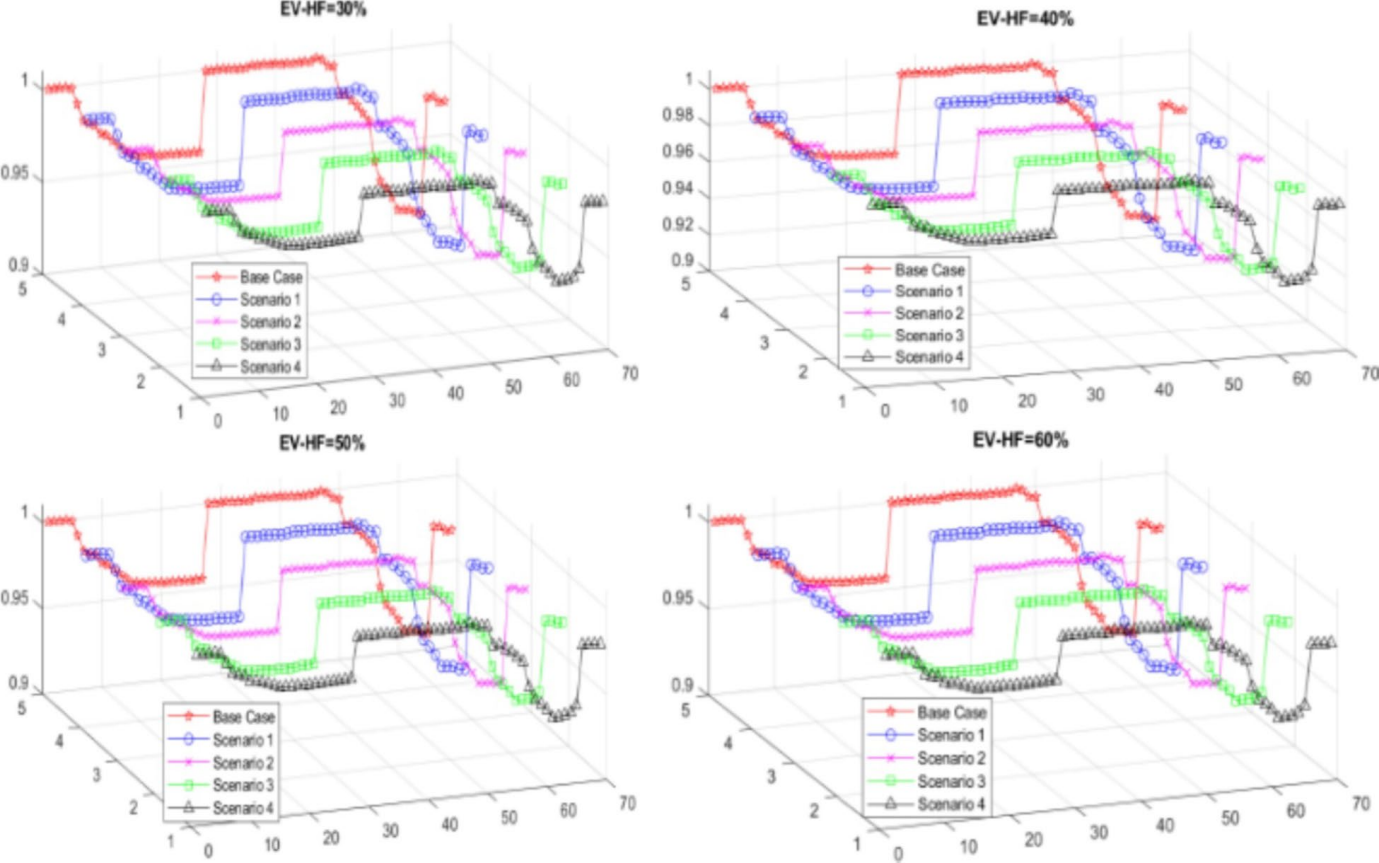
The impact of EV-HF on the voltage profile in various scenarios.

**Fig. 7:**
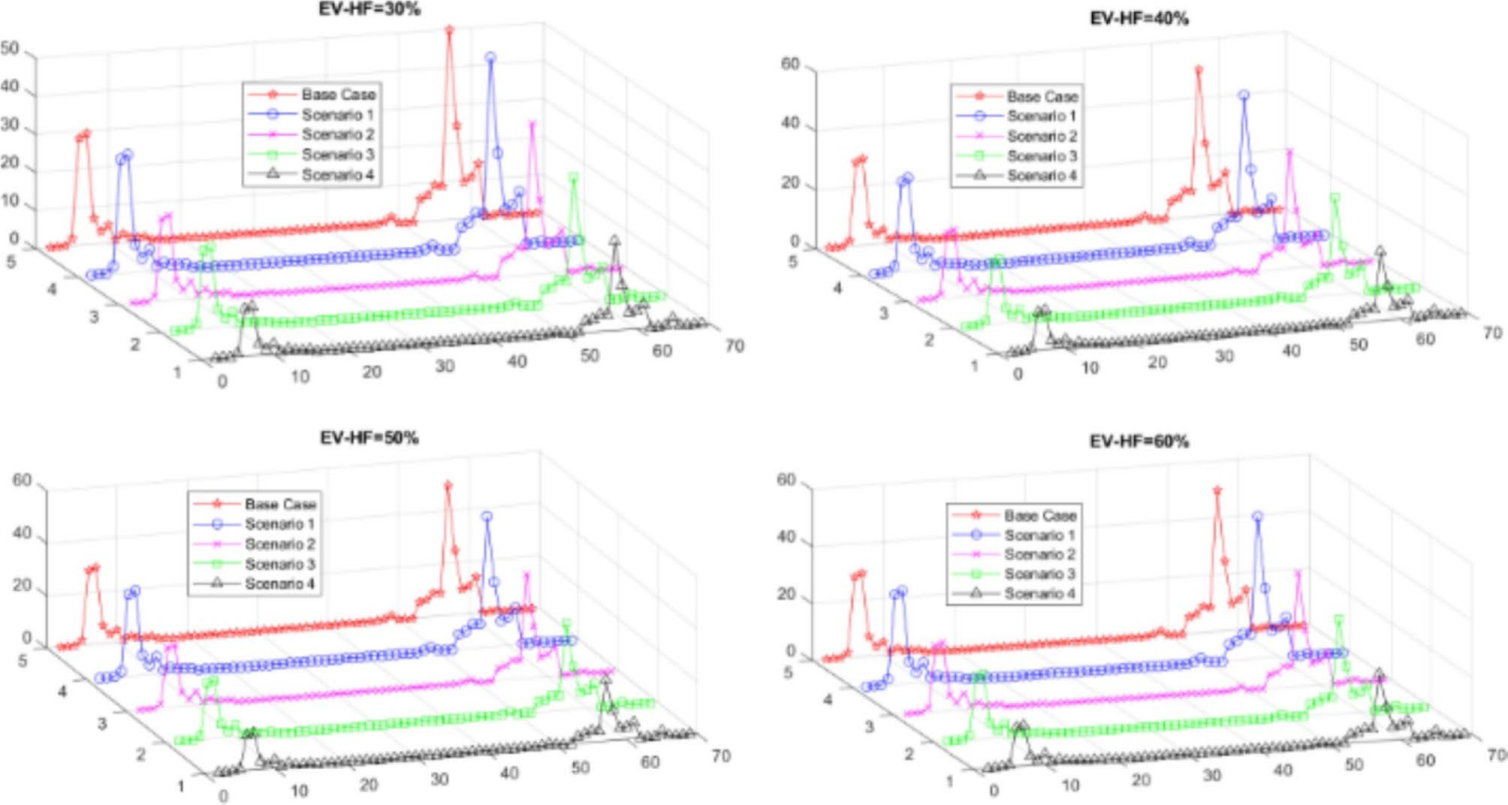
The impact of EV-HF on power loss profiles in various scenarios.

The optimal locations for RDGs in zone two are determined to be at bus number 64. The recommended sizes for this location are 349 kW, 369 kW, 380 kW, and 392 kW for EV-HF levels of 0.3, 0.4, 0.5, and 0.6, respectively, with no additional RDGs needed in other zones, as shown in Fig. 5. This integration reduces active and reactive power losses by approximately 29% for all EV-HF values compared to the base case, resulting in an enhanced voltage profile, as illustrated in Fig. 6. The voltage deviation index is reduced to approximately 0.075 in all cases, while the VSI and minimum voltage improve to approximately 0.74 and 0.928, respectively, as shown in Table 4; Fig. 7. The PV calculation parameters are based on the location of Cairo, Egypt, which is situated at 30.033° latitude and 31.562° longitude, The PV calculations are performed using PVGIS, which takes into account the uncertainty of PV systems^68^.

### Scenario 4: the integration of EVCSs, DSTATCOMs, and RDGs

By optimizing the locations of the EVCSs, DSTATCOMs, and RDGs, this scenario achieves the best performance outcomes. The active and reactive power losses decreased by approximately 30.4% and 27%, respectively, across all EV-HF values compared to those of the base case, as shown in Table 4. The voltage deviation index decreases to approximately 0.073 for different EV-HF scenarios, while the VSI and minimum voltage increase to 0.7724 and 0.93, respectively. The best voltage and power loss profiles across all scenarios are achieved in this scenario, as illustrated in Figs. 6 and 7.

The optimal placement for the DSTATCOM and RDG was determined to be at bus 64. The sizing for the DSTATCOM is specified as 264 kVAR, 272 kVAR, 273 kVAR, and 278 kVAR for EV-HF values of 0.3, 0.4, 0.5, and 0.6, respectively. Correspondingly, the size of the RDG is equal to that of the DSTATCOM but measured in kW.

In summary, the integration of EVCSs, DSTATCOMs, and RDGs significantly enhances the performance of the RDN by reducing power losses and improving voltage stability. These results underscore the importance of the optimal placement and sizing of these components in addressing the challenges posed by increasing EV and renewable energy integration in distribution networks.

**Table 4:** The impact of EV-HF on the objectives in various scenarios.

Scenario	Base	1	2	3	4
P_loss_ (kW)	225	230.88–237.28	155.45–162.98	159.85–159.89	154.11–156.5
Q_loss_ (kVAR)	102.2	105.14–108.39	72.18–75.63	74.84–75.25	72.37–73.85
VSI	0.6868	0.6845 − 0.6821	0.745 − 0.739	0.7363–0.7445	0.742–0.7421
VDI	0.0993	0.1034–0.1076	0.0594–0.0653	0.0723–0.0754	0.0738–0.0761
V_min_ (pu)	0.9092	0.9085 − 0.9077	0.928 − 0.9261	0.9296 − 0.9286	0.9289 − 0.9287
Costs ($)	–	–	$80,750–$100,450	$365,310–$388,080	$275,880–$290,510
Annual saving costs ($)	–	–	$32,600.7–$36,560.6121	$44915.1–$39988.2204	$52,568–$53,495
Payback period (years)	–	–	2.7–3	8.7–9.4	9.8–10.4
Total profit ($)			$714,267.5–$816,515	$747,877.5–$999,705	$1,039,320–$1,052,365

From a technical perspective, integrating EVCSs alone in Scenario 1 resulted in an increase in power loss from 225 kW to between 230.88 kW and 237.28 kW (2.6–5.4%) and an increase in reactive power loss from 102.2 kVAR to between 105.14 kVAR and 108.39 kVAR (2.9–6%). However, Scenario 2, which combines EVCSs with DSTATCOM, significantly reduces these losses, with the power loss decreasing to between 155.45 kW and 162.98 kW (31–27.6%) and the reactive power loss decreasing to between 72.18 kVAR and 75.63 kVAR (29.4–26%).

PV integration alone in Scenario 3 maintains a stable reduction in power loss of approximately 159.85 kW to 159.89 kW (29%) and a decrease in reactive power loss of approximately 74.84 kVAR to 75.25 kVAR (26.8–26.1%). The combined integration of EVCSs, PVs, and DSTATCOMs in Scenario 4 shows the most significant improvements, with the power loss reduced to between 154.11 kW and 156.5 kW (31.5–30.5%) and the reactive power loss reduced to between 72.37 kVAR and 73.85 kVAR (29.2–27.8%).

The voltage stability also improved across the scenarios. VSI increases from 0.6868 to between 0.745 and 0.739 (8.5–7.6%) in Scenario 2. V_min_ increases from 0.9092 p.u. to between 0.928 p.u. and 0.9261 p.u. (2.1–1.9%) in Scenario 2, indicating enhanced voltage stability. Furthermore, the VDI improved from 0.0993 to between 0.0594 and 0.0653 (40.2–34.2%) in Scenario 2.

Economically, Scenario 2, which integrates EVCS and DSTATCOM, presents significant benefits, with total DSTATCOM costs ranging from $80,750 to $100,450 and annual savings between $32,600.7 and $36,560.6, resulting in a payback period of 2.7 to 3 years. Scenario 3, which focuses on PV integration, has installation costs ranging from $365,310 to $388,080, with a payback period of 8.7 to 9.4 years and a total profit over 25 years ranging from $747,877.5 to $999,705. Scenario 4, which combines EVCS, PV, and DSTATCOM, has total costs ranging from $275,880 to $290,510, with annual savings of $52,568 to $53,495, a payback period of 9.8 to 10.4 years, and a total profit over 25 years ranging from $1,039,320 to $1,052,365.

## Conclusion

Integrating EVCSs, DSTATCOMs, and PVs leads to improvements in power loss, voltage stability, and operational efficiency.

### Scenario 1 (EVCS integration alone)

- Power loss increased from 225 kW to 237.28 kW (5.4% increase).
- Reactive power loss increased from 102.2 kVAR to 108.39 kVAR (6.0% increase).

### Scenario 2 (EVCSs with DSTATCOM)

- Power loss decreased to 155.44 kW (31% reduction).
- Reactive power loss decreased to 72.17 kVAR (29.4% reduction).

### Scenario 3 (PV integration alone)

- Power loss reduced to 159.88 kW (29% reduction).
- Reactive power loss reduced to 75.24 kVAR (26.4% reduction).

### Scenario 4 (combined EVCSs, PVs, and DSTATCOMs)

- Power loss reduced to 154.10 kW (31.5% reduction).
- Reactive power loss reduced to 72.36 kVAR (29.2% reduction).

### Voltage stability improvements

- Scenario 2: VSI increased from 0.6868 to 0.7449 (8.5% increase).
- Scenario 4: Vmin increased from 0.9092 p.u. to 0.9286 p.u. (2.1% increase).
- Scenario 2: VDI improved from 0.0993 to 0.0594 (40.2% reduction).

### Economic benefits of technology integration

- Scenario 2 (DSTATCOM costs):
- Costs: $80,750 to $100,450.
- Annual savings: $32,600.7 to $36,560.6.
- Payback period: 2.7 to 3 years.
- Total profit (25 years): $714,267.5 to $816,515.
- Scenario 3 (PV installation costs):
- Costs: $365,310 to $388,080.
- Payback period: 8.7 to 9.4 years.
- Total profit (25 years): $747,877.5 to $999,705.
- Scenario 4 (combined integration):
- Costs: $275,880 to $290,510.
- Annual savings: $52,568 to $53,495.
- Payback period: 9.8 to 10.4 years.
- Total profit (25 years): $1,039,320 to $1,052,365.

Scenario 2 is the best overall option because it achieves significant technical improvements by simply integrating DSTATCOMs, which effectively reduces power loss and reactive power loss. Additionally, Scenario 2 has the advantage of a lower initial cost ($80,750 to $100,450) compared to other scenarios, making it a more affordable solution. The short payback period of 2.7 to 3 years further enhances its appeal, as it allows for quicker financial returns, with total profits ranging from $714,267.5 to $816,515 over 25 years. This combination of technical and economic benefits makes Scenario 2 the most efficient and cost-effective solution.

Conclusion: Strategic deployment of EVCSs, PVs, and DSTATCOMs, along with continuous performance monitoring, is essential for achieving an efficient, stable, and cost-effective power distribution network.

### Future work

While this study has effectively demonstrated the benefits of integrating electric vehicles (EVs), DSTATCOM, and renewable energy sources using the Hippopotamus Optimization Algorithm, several avenues for future research remain. One critical area is the consideration of energy storage systems (ESS). Integrating ESS with renewable energy sources is crucial for mitigating the mismatch between stochastic renewable generation and demand. Proper planning and optimization of ESS would provide more reliable power, reduce the need for grid upgrades, and enhance overall system stability, particularly during periods of low renewable generation or high demand surges^54^.

Another important consideration for future studies is the incorporation of EV-specific constraints—such as driving ranges, charging durations, user behavior, and EV penetration rates—into the placement and sizing of charging stations. These constraints significantly impact the effectiveness and practicality of EV charging infrastructure.

## Data Availability

The datasets generated during the current study are available from the corresponding author upon reasonable request.
